# *Korean Journal of Women Health Nursing* is indexed in Scopus and stepping closer to international connectivity

**DOI:** 10.4069/kjwhn.2021.06.16

**Published:** 2021-06-23

**Authors:** Sue Kim

**Affiliations:** Mo-Im Kim Nursing Research Institute, College of Nursing, Yonsei University, Seoul, Korea

I am delighted to announce that the *Korean Journal of Women Health Nursing* (KJWHN) received notice from the Scopus Content Selection and Advisory Board (CSAB) on April 13, 2021, that the journal will be included in the Scopus database.

This recognition from Scopus builds on the dedication and tireless work of prior editors-in-chief and editorial board members and signals the continuous work needed for KJWHN to continue its growth while striving for international connectivity.

The journal was launched in March 1995 under its previous title, the *Journal of Korean Women's Health Nursing Academic Society*, publishing eight articles in the first issue. Till June 2012 the journal has been published under the current title. Throughout the past 25 years, KJWHN has been “The” scholarly platform for sharing among Korean nurses in maternal and women’s health for nurses in clinical practice, nurse researchers, and students alike. KJWHN was an early pioneer among Korean nursing journals in terms of offering open access since 2008 and being included in CINAHL in 2009, as well as the Directory of Open Access Journals (DOAJ) in 2013.

The following timeline illustrates our path of seeking and achieving Scopus status, which may help other journals: In 2013, the first application to Scopus was submitted. A few months later, the editorial office received notice of rejection with the following comments: “Citedness is below expectations. There is no international diversity among authors. Some part of reference listing is inconsistent with the author instructions.”

Acting on these comments, the editorial board was expanded and editors’ efforts to advertise the journal and encourage international authors resulted in a manuscript submission from Turkey, which was eventually published.

In August 2015, the second application was submitted, and another letter was received in November 2016, citing the following rejection reasons: “Some part of reference listing is inconsistent with the author instructions. There is no international diversity among editors and low citation activity on editorial board level.”

In response, the editorial board worked over the past 4 years to overcome the shortcomings before reapplying in January 2021. It adopted an internal system to screen for inconsistencies with author instructions, including the reference list and conducted regular training of editorial board members. Furthermore, since 2020, KJWHN has invited a professional manuscript editor to ensure consistency with author instructions. Regarding the second limitation, the editorial board and international advisory board were restructured and expanded. While KJWHN had six international advisory board members from three countries in 2016, this was expanded to nine international members from five countries across three continents as of January 2021. These international members are active professionals in their field, and are contributing to the journal via peer-review as well as writing invited papers for the journal. Finally, for citation activity, editorial board members’ academic activity was made available on our website via Scopus Author ID and ORCID links. In addition to addressing the specific comments, editors have worked diligently to improve the journal according to international standards. The journal went through three rounds of revising author guidelines for clarity since 2020, created a statement on *Principles of Transparency and Best Practice in Scholarly Publishing* version 3 [1], updated the publication ethics content to reflect international academic publishing recommendations, such as the Committee on Publication Ethics (COPE), and formally enlisted the expertise of an Ethics Editor. The journal moved to a new English-based online submission system that facilitates journal management for authors and reviewers from Korea and abroad (https://submit.kjwhn.org). Our new English-based website continues to support full open access (https://kjwhn.org), in addition to translations powered by Google in 80 languages and metrics information. Information on funding sources was made available from 2020.

After implementing these changes, in January 2021, the third application to Scopus was submitted. The acceptance letter from CSAB, received on April 13, 2021, included the following comments: “The previous and current editorial teams are to be commended on the development which has led to extended citations in international research journals. Many papers have been cited. The development of the ethical standards throughout is very clear and in keeping with both COPE and Scopus guidance. The use of the COPE et al.’s 2018 Principles of Transparency guidance was especially of value and demonstrates an ongoing attention to publishing practice and ethical content of the journal.”

While I am delighted the journal is now accepted into the Scopus database, I realize this is a timely opportunity and significant time of reflection for KJWHN to consider how to potentiate its value for authors and reach a wider international readership in the coming future. In this line, I want to add a few notes on how the journal seeks to further improve itself over the next few months.

Citations are a constant challenge for all journals. While the adage for academia is ‘publish or perish,’ for journals this seems to have become what I shall term ‘be cited to survive and thrive.’ The total cites in the Scopus database have increased annually as follows: 71 in 2017, 96 in 2018, 123 in 2019, and 125 in 2020. The majority of citations, however, are from Korean journals in Scopus, both nursing and others (Figure 1). Also, most citations are from Korea (330 documents), followed by the United States (51 documents) and Iran (Figure 2). Now that references of the journal will be added to the Scopus database, I look forward to journal metrics improving and greater opportunities to increase international connectivity.

I will continue to examine our policies to be up-to-date with international scholarly publishing trends and directions. From the June issue, several changes were implemented: Firstly, data sharing is now possible through Harvard Dataverse (https://dataverse.harvard.edu) for authors who wish to share data for transparency and replicability. Also, ascribing to internationally accepted reporting guidelines, e.g., those suggested by the Equator network (https://www.equator-network.org/), is being applied. In addition to these updates to the author guidelines, the following will be applied from the 2021 September issue: Manuscript types for submission have been expanded and requirements for each type have been clarified (Table 1).

Readers may notice that the majority of articles are in English in this issue. Some Korean journals have chosen to switch to publishing fully in English, such as the *Archives of Plastic Surgery* (APS) and more recently, *Child Health Nursing Research*, a domestic nursing journal. In the case of APS, since switching to English-only manuscripts in 2012, publication metrics improved and it was accepted for the Scopus database in 2013 and included in the Web of Science Core Collection in 2016 [2,3]. Indeed, the exponential growth over a short period of roughly 4 years following the English-only policy, appears to suggest an exemplary pathway for other Korean journals. Considering the criticism about structural bias towards Western, English-based research among global databases such as Web of Science and Scopus [4], and the inherent bias of impact factors against non-English language and culture-bound health fields [5], perhaps this is indeed a tested, recommendable strategy. However, not all journals can immediately focus on becoming international-based journals publishing only in English, nor should this be the automatic ‘gold standard’ for all. Publishing in the native language is justified and necessary, especially in cases when local applications of the research or domestic impact of the articles are more relevant and important for the national and/or regional community [6]. An example is the series of method papers on measurement tool reliability and validity, that are purposively being published in the Korean language since our 2021 March issue [7,8], as a means of clarifying Korean terminology and conceptual understanding for our Korean readers.

Nevertheless, an analysis of natural science journals from several countries publishing in English or other languages, reported that English publications had a higher number of citations than those published in other languages, even when controlling for the effect of the journal, year of publication, and paper length [6]. It is worthy to consider the authors’ interpretations that English articles have the advantage of reaching a larger audience, may avoid limitations in scientific perspective, and can be conducive for potential scientific collaboration. As such, journals often have to weigh serving the needs of domestic readers and scholars through native language publications, while expanding English publications for the sake of increasing international interest and citations. KJWHN will continue to value Korean-language submissions in order to serve the Korean scholarly community, while it will simultaneously focus on offering quality English articles, both from within and abroad. This is in line with KJWHN’s mission to ultimately become a top-tier journal for women’s health nursing in Asia.

The success of our efforts will largely depend on a solid number of submissions. I invite authors to submit their work, especially systematic review and meta-analysis papers, experimental design studies, exploratory qualitative research, and methodology manuscripts. For descriptive and correlational research design manuscripts, I encourage authors to include a description of the theoretical or conceptual framework used to ground their quantitative research, which has been emphasized as good practice [9].

The present achievements of KJWHN have been possible through the support and cooperation of society members and readers worldwide. I truly appreciate all of them for their contribution. To promote the journal to an internationally top-ranking one, the continuous attention of nursing scholars in Korea and worldwide are essential. The journal will continue to promote maternal and women’s health nursing by following international publishing standards and thoroughly editing and publishing manuscripts.

## Figures and Tables

**Figure 1.**
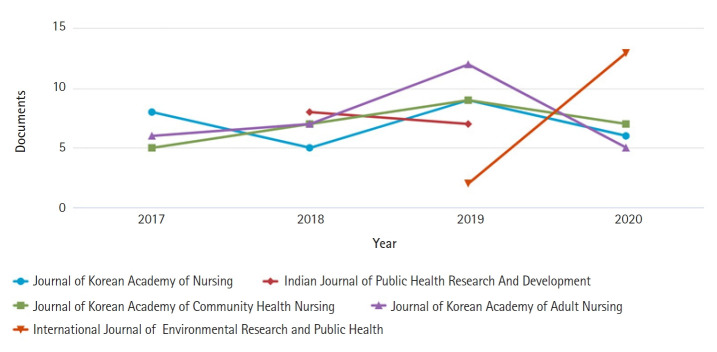
Documents per year by cited source for the *Korean Journal of Women Health Nursing* per Scopus database.

**Figure 2.**
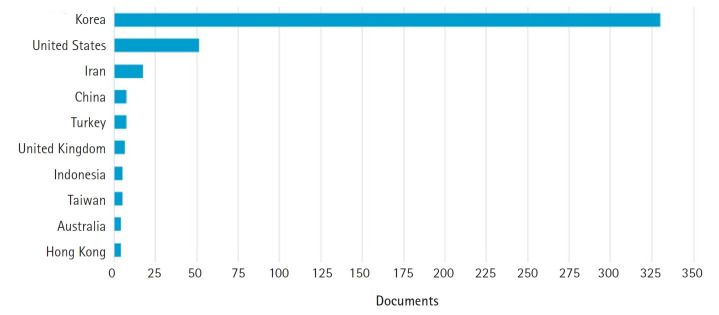
Citing documents by country or territory for the *Korean Journal of Women Health Nursing* per Scopus database.

**Table 1.** Recommended maximums for articles submitted to the *Korean Journal of Women Health Nursing*

Publication type	Abstract (word count)	Text (word count)^a)^	References	Tables & figures	Invited or unsolicited
Original articles	250	5,000	No limit	6	Unsolicited
Review articles	250	8,000	No limit	6	Invited or unsolicited
Invited papers	Optional (250)	8,000	30	10	Invited
Issues and Perspectives	None	2,000	20	10	Invited
Special essays	None	3,000	20	10	Invited
Editorials	None	2,500	10	5	Invited
Letter to the editor	None	1,000	10	3	Unsolicited
In reply	None	1,000	10	3	Invited

a) Maximum number of words excludes the abstract, references, tables, and figure legends.

Above limitations are negotiable. If more word count or the number of figures and tables are required, authors can contact the editor-in-chief.
